# Mechanical wrist traction as a non-invasive treatment for carpal tunnel syndrome: a randomized controlled trial

**DOI:** 10.1186/s13063-017-2208-9

**Published:** 2017-10-10

**Authors:** Margreet Meems, Viola Spek, Willem J. Kop, Berend-Jan Meems, Leo H. Visser, Victor J. M. Pop

**Affiliations:** 1 Department of Medical and Clinical Psychology, Centre of Research on Psychological and Somatic disorders (CoRPS), Tilburg University, PO Box 90153, 5000 LE Tilburg, The Netherlands; 2 Division of Neurology, VieCuri Medical Center, PO Box 1926, 5900 BX Venlo, The Netherlands; 3 Division of Neurology, Elisabeth-TweeSteden Hospital, PO Box 90151, 5000 LC Tilburg, The Netherlands

**Keywords:** Boston carpal tunnel questionnaire, Carpal tunnel release surgery, Carpal tunnel syndrome, Mechanical traction

## Abstract

**Background:**

Carpal tunnel syndrome (CTS) is a common, compressive nerve-entrapment disorder with symptoms of numbness, paresthesia, and pain. Carpal tunnel release surgery is the only known long-term effective treatment. However, surgery is invasive and up to 30% of patients report recurrence or persistence of symptoms or suffer from post-surgical complications. A promising non-surgical treatment for CTS is mechanical wrist traction. The purpose of this study was to evaluate clinical outcomes following mechanical traction in patients with CTS compared to care as usual.

**Methods:**

Adult patients (*N* = 181, mean age 58.1 (13.0) years, 67% women) with electrodiagnostically confirmed CTS were recruited from an outpatient neurology clinic in the Netherlands between October 2013 and April 2015. After baseline assessments, patients were randomized to either the intervention group (12 treatments with mechanical traction, twice a week for a period of 6 weeks) or “care as usual”. The main clinical outcome measure was surgery during 6 months’ follow-up. In addition, symptom severity was measured using the Boston Carpal Tunnel Questionnaire (BCTQ) at baseline, 3, and 6 months’ follow-up. Baseline characteristics and severity of CTS symptoms at follow-up were compared between the intervention and care-as-usual groups using a *t* test and *χ* ^2^ tests. Time to event (surgery) between the groups was analyzed using Kaplan-Meier survival analysis and Cox proportional hazards analysis.

**Results:**

The intervention group had fewer surgeries (28%) compared to the care-as-usual group (43%) during follow-up (χ^2^_1_ = 4.40, *p* = .036). Analyses of the survival curves revealed a statistically significant difference between the groups over time (log-rank test *χ* ^2^_1_ = 6.94, *p* = .008). At 6 months’ follow-up, symptom severity and functional status scores had significantly decreased from baseline in both groups (*p* < .001) and the improvements did not differ between the two groups.

**Conclusions:**

Mechanical traction is associated with fewer surgical interventions compared to care as usual in CTS patients. Reductions in patient-reported symptoms at 6 months’ follow-up was similar in both groups. The long-term effects of mechanical traction require further evaluation.

**Trial registration:**

ClinicalTrials.gov, ID: NL44692.008.13. Registered on 19 September 2013.

## Background

In recent years, concerns have been raised about the large number of invasive treatments performed every year [1]. In some cases, initial conservative treatment may be more cost-effective and preferred by the patient [2]. This especially applies to carpal tunnel syndrome (CTS). CTS is a compressive nerve disorder in which the median nerve is compressed in the carpal tunnel [3, 4]. It is very common; the prevalence of CTS in the general population in the United States and the Netherlands is 4–5% [5–8]. The compression leads to numbness, paresthesia, and pain, especially in the first three digits and the radial side of the ring finger, areas which are innervated by the median nerve. Symptoms are typically worse at night [3]. CTS can be diagnosed using electrodiagnostic testing to detect slowing of conduction velocity that results from median nerve compression-related damage and dysfunction of the myelin sheath [9]. Treatment options to relieve symptoms are either surgical or non-surgical. Non-surgical, less-invasive treatment options include oral non-steroidal drugs, corticosteroids (injections), splinting, exercise, and mobilization interventions [10–14]. There is only short-term or limited evidence of benefit for these interventions. Many (non-steroidal) drugs are not significantly superior to placebo [12]. Local corticosteroid injections provide considerable symptom relief [10, 11], but seem to primarily suppress CTS symptoms and the treatment effect diminishes over time [10, 15]. There is only limited evidence for the effectiveness of splinting, exercise, and mobilization interventions [13, 14].

Carpal tunnel release surgery is the only known treatment option with long-term positive effects [16]. Evidence suggests that surgery is a more effective treatment for CTS than conservative treatment (splinting or steroid injections) [16–18]. However, surgery is associated with several disadvantages: some patients suffer from sustained surgery-related pain, hand weakness or complications from surgery [19]. Patient satisfaction rates of carpal tunnel release surgery vary between 70 and 80% [7, 20–23]. The remaining 20–30% report persistence or recurrence of CTS symptoms or suffer from complications [21, 22]. The reported frequency of re-operation rate is between 3 and 12% [21]. Therefore, there is a clear need for an alternative, preferably non-invasive, therapy for CTS.

A promising non-surgical treatment for CTS is mechanical wrist traction. This intervention involves repeated traction movements to the wrist in different positions using gravitational force. Brunarski et al. [24] described four case studies using mechanical traction that showed promising results. In an observational study among 78 CTS patients, treatment with mechanical traction resulted in a success rate of 70% immediately post treatment [25], and 60% after 2-year follow-up [26]. However, no randomized controlled trial (RCT) has been performed to show clinical evidence for the effectiveness of mechanical traction as compared to “care as usual” (surgical and non-surgical interventions).

The purpose of this study was to evaluate the impact of mechanical traction in patients with CTS compared to care as usual using an RCT. The main clinical outcome of the study was surgery for carpal tunnel release during 6 months’ follow-up. We also examined differences between mechanical traction vs. usual care on the decrease in symptom severity and hand function problems at follow-up.

## Methods

### Participants

Patients diagnosed with CTS were recruited from the outpatient neurology clinic of VieCuri Medical Center in Venlo and Venray, the Netherlands between October 2013 and April 2015. Adult men and women (aged 18–80 years) who were diagnosed with CTS by means of electrodiagnostic testing were invited to participate in the study. Electrodiagnostic values were considered abnormal if there was a difference greater than 0.5 ms on distal sensory latency (DSL) between the ulnar and median nerves in digit IV or between the radial and median nerves in digit I, a distal motor latency (DML) greater than 3.7 ms across the wrist to digit I of the median nerve (measured in the abductor pollicis brevis), or a difference greater than 0.4 ms in the median nerve across the wrist compared to the palmar branch of the median nerve to digit III. The criteria used for diagnosis were at least two abnormal measures [9].

**Fig. 1:**
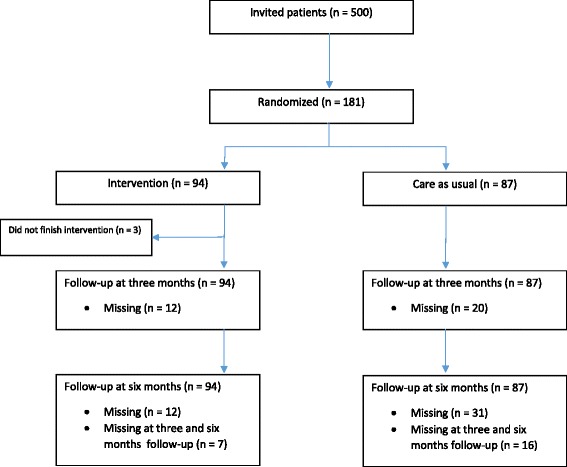
Flow chart of inclusions

Patients with a previous history of CTS surgery were excluded as well as those with insufficient understanding of the Dutch language. In addition, patients who were diagnosed with another known (rare) cause of neuropathy, or who suffered from a severe psychiatric disorder, such as personality disorder, schizophrenia or bipolar disorder, were also excluded. During the visit to the outpatient clinic, the neurologist provided eligible patients with oral and written information about the study. In total, 500 eligible patients were invited of whom 181 patients agreed to participate and were randomized to mechanical traction intervention or care as usual. The main objections to participation were lack of time and transport. The eligibility of included patients was double-checked and full written informed consent was obtained, including access to patient medical record forms. Figure 1 presents a flow chart of participant inclusions, treatment conditions, and data availability.

### Procedure

After inclusion, patients were interviewed by the research staff (MM), during which they also answered a set of paper-and-pencil questionnaires. Demographic variables and lifestyle habits were documented.

After the baseline interview, the 181 patients were randomized into two groups: the traction intervention (*n* = 94) or care as usual (*n* = 87). The randomization procedure is described in detail elsewhere [27]. As shown in Table 1, the groups were comparable on demographic and clinical variables and the slight imbalance in the group sizes resulted from the randomization procedure, which was based on a-priori group allocation of the first 200 participants. The patients filled out questionnaires at baseline and at 3 and 6 months after inclusion, either through an email invitation online or a paper-and-pencil version. There were significantly more dropouts in the care-as-usual group (36%, *n* = 31) compared to the intervention group (13%, *n* = 12, *χ* ^2^_1_ = 13.0, *p* < .001).

**Table 1:** Baseline characteristics of the sample (*n* = 181)

	Total (*n* = 181)		Intervention (*n* = 94)		Care as usual (*n* = 87)		*p*	
	Mean (*SD*)	*n* (%)	Mean (*SD*)	*n* (%)	Mean (*SD*)	*n* (%)	*t*	*X* ^2^
Demographic features								
Age (in years)	58.1 (13.0)		59.0 (12.4)		57.2 (13.7)		.339	
Sex								.454
Male		59 (32.6)		33 (35.1)		26 (29.9)		
Female		122 (67.4)		61 (64.9)		61 (70.1)		
Educational level								.809
Low		149 (82.3)		78 (83.0)		71 (81.6)		
High		32 (17.7)		16 (17.0)		16 (18.4)		
Marital status								.560
With partner		138 (76.2)		70 (74.5)		68 (78.2)		
CTS-related								
Duration of complaints								.212
< 3 years		136 (75.4)		67 (71.3)		69 (79.3)		
> 3 years		45 (24.9)		27 (28.7)		18 (20.7)		
Dominant hand involved?								.950
No		35 (19.3)		19 (20.2)		16 (18.4)		
Yes		53 (29.3)		27 (28.7)		26 (29.9)		
Both hands		93 (51.4)		48 (51.1)		45 (51.7)		
Direct relative with CTS		54 (29.8)		28 (29.8)		26 (29.9)		.989
Paid hand labor?								.605
No		113 (62.4)		57 (60.6)		56 (66.4)		
Heavy		68 (37.6)		37 (39.4)		31 (35.6)		
SSS score	2.86 (0.77)		2.89 (0.80)		2.82 (0.74)		.589	
FSS score	2.34 (0.89)		2.39 (0.92)		2.28 (0.86)		.374	
BCTQ score	2.64 (0.76)		2.68 (0.79)		2.59 (0.72)		.421	
Lifestyle habits								
Smoking		29 (16.0)		16 (17.0)		13 (14.9)		.703
Alcohol		43 (23.8)		24 (25.5)		19 (21.8)		.560
BMI	28.8 (5.08)		29.2 (5.24)		28.4 (4.89)		.296	

*BMI* Body Mass Index, *CTS* carpal tunnel syndrome, *BCTQ* Boston Carpal Tunnel Questionnaire, *FSS* Functional Status Scale, *SD* standard deviation, *SSS* Symptom Severity Scale

#### Intervention: Phystrac mechanical traction therapy

Patients in the intervention group received 12 treatment sessions (twice a week for a period of 6 weeks) with the Phystrac mechanical traction device (type GR 10). The Phystrac provides mechanical traction to the wrist using weights of between 1 and 18 kg. One session takes 10 to 15 min per affected hand. The weight was set at 5 kg for women and 7 kg for men during the first session. Every following session, the weight was increased with 1 kg for women and 2 kg for men until 10 kg for women or 13 kg for men, or until the mechanical traction became uncomfortable for the patient. Twelve treatments are considered sufficient for most patients. When 12 treatments with mechanical traction were not effective at reducing CTS symptoms, participants could subsequently receive care as usual.

#### Control group: “care as usual”

The control group received care as usual, which meant that participants received regular treatment from their usual health care provider. Patients adopted an expectant approach or received treatment in the form of a wrist splint, local corticosteroid injections or carpal tunnel release surgery. Forms of treatment received in both groups were documented during the full length of the study using questionnaires and checking the medical records.

### Outcome measures

The main outcome variable was whether patients received surgery during follow-up, which was derived from the patient medical records. Self-reported functional status and symptom severity were measured using the Boston Carpal Tunnel Questionnaire (BCTQ) [28, 29]. The BCTQ is a disease-specific questionnaire referring to a typical 24-h period in the past 2 weeks. It consists of two scales: the Symptom Severity Scale (SSS) and the Functional Status Scale (FSS). The SSS consists of 11 questions about symptom severity, while the FSS consists of eight daily activities which are rated based on degree of difficulty. The SSS and the FSS are rated on a five-point scale. Both scales result in mean scores between 1 and 5, where greater impairment is represented by higher scores. The total BCTQ score is calculated as the mean of all the items. The BCTQ is responsive to clinically relevant change and is, therefore, an appropriate measure for treatment outcome [28]. It has been validated and is used in multiple studies to assess improvement in CTS symptoms over time [16], also in the Netherlands [7, 15].

### Statistical analyses

Data are presented as mean ± standard deviation (SD) or percentages. An independent samples’ *t* test and *Χ* ^2^ tests were used to compare characteristics between groups. Time-to-first-event (surgery) curves were displayed for the intervention and care-as-usual groups using Kaplan-Meier analysis. Cox proportional hazards analysis was used with group, age, sex, pre-enrollment symptom duration, dominant hand involved, and baseline BCTQ score as predictor variables. The proportional hazards assumption was visually checked based on the survival curves and the log minus log survival vs. log of survival time curves for the different covariates. Analyses were performed using an intention-to-treat approach. To avoid loss of data, missing data were imputed using multiple imputation. Statistical analyses were performed using the Statistical Package of Social Science (SPSS, 22.0).

## Results

Baseline characteristics of the sample are presented in Table 1. The mean age was 58.1 (13.0) years, 67% were women and 25% had had CTS complaints for longer than 3 years. The care-as-usual and intervention groups did not differ based on any of the baseline characteristics.

We also compared the patients included in the trial (*n* = 181) to all eligible patients on age, sex, and affected hand. Patients who participated in the trial were older than the overall group of eligible patients (58.1 (13.0) and 54.8 (14.4), respectively; *p* = .011). Moreover, within the included patients, we compared baseline characteristics of the completers with those of the dropouts at 6 months’ follow-up. The patients who dropped out were significantly younger than the completers in the whole study population (53.7 (14.9) and 59.5 (12.1) years, respectively; *p* = .010), as well as within the intervention (*p* = .036), but not within the care-as-usual group (*p* = .157).

### Effects of mechanical traction on surgery during six months follow-up

**Fig. 2:**
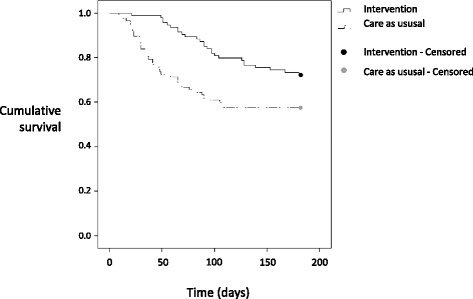
Kaplan-Meier survival curves. Analysis of the number of days after randomization until occurrence of surgery for the intervention and care-as-usual groups (log-rank test *χ* ^2^_1_ = 6.94, *p* = .008)

At 6 months’ follow-up, 26 (28%) patients in the intervention group had had surgery, compared to 37 (43%) in the care-as-usual group (*χ* ^2^_1_ = 4.40, *p* = .036). Kaplan-Meier survival curves showed significant group differences over time (Fig. 2; log-rank test *χ* ^2^_1_ = 6.94, *p* = .008). Time to surgery was shorter (median = 41 days) in the care-as-usual group than in the intervention group (median = 90 days). The proportional hazards assumption was verified. Cox proportional hazards analysis revealed a significantly higher rate of surgery during 6 months’ follow-up in those receiving care as usual (hazard ratio (HR) = 2.27, 95% CI = 1.35–3.80), as well as in those with a symptom duration of more than 3 years (HR = 1.89, 95% CI = 1.11–3.24), adjusted for age, sex, dominant hand involved, and BCTQ score at baseline.

### Symptom status at follow-up

At 6 months’ follow-up, symptom severity and functional status scores did not differ between the intervention and care-as-usual group (Table 2). When comparing the change in scores from baseline to 6 months’ follow-up (paired samples *t* test) based on intention-to-treat, the BCTQ scores decreased significantly both in the intervention (*p* < .001) as well as the care-as-usual group (*p* < .001).

**Table 2:** Comparison of carpal tunnel syndrome (CTS) symptom scores at 6 months’ follow-up between the intervention and care-as-usual groups

	Mean (SE) score intervention group (*n* = 94)	Mean score (SE) care as usual group (*n* = 87)	*t*	*p*
SSS	1.84 (0.10)	1.89 (0.14)	− .32	.747
FSS	1.75 (0.09)	1.75 (0.09)	− .07	.947
BCTQ	1.80 (0.08)	1.84 (0.08)	− .31	.756

*BCTQ* Boston Carpal Tunnel Questionnaire, *FSS* Functional Status Scale, *SE* standard error, *SSS* Symptom Severity Scale

## Discussion

Patients who received care as usual had a 2.3-fold risk of receiving carpal tunnel release surgery compared to patients who were treated with mechanical traction during the follow-up period. Moreover, the symptom severity had decreased significantly from baseline to follow-up in both groups, suggesting that not having received surgery did not result in a persistence of CTS-related symptoms.

To date, this is the only RCT evaluating the effectiveness of mechanical traction. In 2004, Brunarski et al. [24] published a series of four case studies in which patients with CTS received mechanical traction. In all four cases, symptoms improved both subjectively and objectively (measured using electrodiagnostic studies). In an observational study among 78 patients, symptoms reduced significantly after mechanical traction immediately post treatment (*p* < .01) [25]. After 2 years’ follow-up, 60% reported a reduction on the SSS subscale of the BCTQ compared to baseline and only 18% had had surgery [26]. A few studies have compared other non-surgical treatments with surgery. Two RCTs comparing steroid injections to surgery reported conflicting results; one in favor of steroid injections [30] and the other in favor of surgery [18]. Jarvik et al*.* [16] compared surgery to non-surgical treatment (anti-inflammatory drugs, hand therapy, and ultrasound therapy) in 116 patients. At 6 months’ follow-up, patients in the surgery group (*n* = 57) had significantly lower scores on the subscales SSS (−0.42) and the FSS (− 0.46) of the BCTQ compared to patients in the non-surgical treatment group (*n* = 59), but the clinical relevance of the difference was only modest. In a study by Gerritsen et al. [8], surgery was more effective than splinting. However, they did not include patients with very mild or very severe symptoms. Altogether, evidence regarding the effectiveness of non-surgical compared to surgical treatment is limited and has provided conflicting results.

In line with the current study, Baker et al. [31] reported that an orthosis and stretch intervention was inversely related to progression to surgery at 6 months. In the current study, over 35% fewer patients underwent surgery compared to the care-as-usual group. Many patients do not prefer surgery when there is another non-invasive treatment available. There are several reasons for patients, or physicians, to choose conservative treatment, including symptom severity, age, patients’ perception of the efficacy of a certain treatment, insurance, patients’ educational level, pregnancy status (during which CTS is highly prevalent) and the presence of comorbidities [32–35].

The median time to surgery was longer in the intervention group compared to the care-as-usual group, meaning that surgery was delayed for approximately the duration of the intervention. Therefore, it is not likely that a substantial number of patients will have had surgery after 6 months in the intervention group compared to the care-as-usual group. Moreover, both survival curves in Fig. 2 regress to a horizontal line, which also suggests not many people receive surgery after 6 months in both groups.

The characteristics of CTS patients in the present investigation are comparable to the CTS patient sample at an outpatient neurology clinic (*n* = 116) in the study by Jarvik et al. [16], based on age, gender, percentage of patients with bilateral symptoms and mean SSS score (2.86 vs. 2.98, respectively). Compared to the population of patients with electrodiagnostically confirmed CTS in the study by Gerritsen et al. [8], our study population is slightly older (58 vs. 49 years), we included less women (67% vs. 76%) and patients with more severe symptoms (mean SSS 2.9 vs. 2.5). Gerritsen et al., however, applied more specific exclusion criteria, excluding patients with diabetes, which could explain the difference.

Patients included in the study did not differ from eligible patients at the outpatient neurology clinic based on the distribution of gender and affected hand; however, they were slightly older. The main objections to participation were long traveling distance to the clinic and lack of time. Older people often have more time, because they are less likely to have young children or a full-time job. In the care-as-usual group, there was a significantly higher dropout rate compared to the intervention group, which is a common limitation of randomized controlled trials: participants may be disappointed to be randomized to the control group and feel less motivated to continue [36].

A limitation of the study is the lack of discrimination between hands in bilaterally affected patients. Other studies only included the most severely affected hand [37] or assessed both hands separately [38]. However, it is often difficult for patients to discriminate between hands when assessing symptom severity, and only including one hand in bilaterally affected patients was not preferable for ethical and practical reasons. Another limitation is that the response to intervention was based on the patients’ symptoms perception, not by an objective measure, such as electrodiagnostic testing. However, electrodiagnostic testing is not sensitive enough to clinical change following treatment: after surgery, nerve conduction improves, but only moderately correlates to patient-reported improvement [39]. Patient-reported outcomes are considered superior in evaluating treatment effect and are used in most studies evaluating treatment effectiveness and the BCTQ is a highly validated self-reporting symptom questionnaire which is commonly used in clinical practice to evaluate changes of symptoms after treatment [7, 16, 28]. Lastly, complete data on other forms of treatment received by patients (other than mechanical traction or surgery) was not available. Of the 82 participants who completed in the intervention group, three patients had received a corticosteroid injection and 14 patients used a wrist splint at follow-up. Of the 56 participants who completed in the care-as-usual group, three patients had received a corticosteroid injection and 11 used a wrist splint. A strength of this study is the selection of the control group. Patients in the control group received care as usual, and hence we compared the intervention to standard care and not to a specific control treatment. This design leads to more generalizable results.

## Conclusions

Treatment of CTS by means of mechanical traction can possibly prevent progression of symptoms requiring surgery within 6 months in CTS patients. Because up to 30% of patients who received surgery report (new) CTS symptoms at longer follow-up (1 to 2 years), a longer period of observation is needed to compare the long-term effect of mechanical traction to care as usual (including surgery). The mechanism for the effectiveness of mechanical traction is still unclear. We expect that traction improves blood microcirculation, reduces edema in the synovial tissue and, therefore, reduces pressure in the carpal tunnel [14, 24]. Future studies should focus on what the possible working mechanism is of mechanical traction. The clinical relevance of the current study is that by introducing a new non-invasive treatment of CTS, different subgroups of CTS patients might be identified who will benefit from mechanical traction. This approach may result in a substantial reduction in the number of surgeries with similar patient-reported symptoms. Mechanical traction may prove to be a more cost-effective intervention for CTS than surgery. Moreover, the current study contributes to the current discussion regarding the critical evaluation of the actual benefit of invasive interventions in general in comparison to more conservative approaches.

## Abbreviations

BCTQ: Boston Carpal Tunnel Questionnaire
CTS: Carpal tunnel syndrome
DML: Distal motor latency
DSL: Distal sensory latency
FSS: Functional Status Scale
RCT: Randomized controlled trial
SSS: Symptom Severity Scale
